# The SMC Complex MukBEF Recruits Topoisomerase IV to the Origin of Replication Region in Live *Escherichia coli*

**DOI:** 10.1128/mBio.01001-13

**Published:** 2014-02-11

**Authors:** Emilien Nicolas, Amy L. Upton, Stephan Uphoff, Olivia Henry, Anjana Badrinarayanan, David Sherratt

**Affiliations:** Department of Biochemistry, University of Oxford, Oxford, United Kingdom

## Abstract

The *Escherichia coli* structural maintenance of chromosome (SMC) complex, MukBEF, and topoisomerase IV (TopoIV) interact *in vitro* through a direct contact between the MukB dimerization hinge and the C-terminal domain of ParC, the catalytic subunit of TopoIV. The interaction stimulates catalysis by TopoIV *in vitro*. Using live-cell quantitative imaging, we show that MukBEF directs TopoIV to *ori*, with fluorescent fusions of ParC and ParE both forming cellular foci that colocalize with those formed by MukBEF throughout the cell cycle and in cells unable to initiate DNA replication. Removal of MukBEF leads to loss of fluorescent ParC/ParE foci. In the absence of functional TopoIV, MukBEF forms multiple foci that are distributed uniformly throughout the nucleoid, whereas multiple catenated *ori*s cluster at midcell. Once functional TopoIV is restored, the decatenated *ori*s segregate to positions that are largely coincident with the MukBEF foci, thereby providing support for a mechanism by which MukBEF acts in chromosome segregation by positioning newly replicated and decatenated *ori*s. Additional evidence for such a mechanism comes from the observation that in TopoIV-positive (TopoIV^+^) cells, newly replicated *ori*s segregate rapidly to the positions of MukBEF foci. Taken together, the data implicate MukBEF as a key component of the DNA segregation process by acting in concert with TopoIV to promote decatenation and positioning of newly replicated *ori*s.

## IMPORTANCE

Mechanistic understanding of how newly replicated bacterial chromosomes are segregated prior to cell division is incomplete. In this work, we provide *in vivo* experimental support for the view that topoisomerase IV (TopoIV), which decatenates newly replicated sister duplexes as a prelude to successful segregation, is directed to the replication origin region of the *Escherichia coli* chromosome by the SMC (structural maintenance of chromosome) complex, MukBEF. We provide *in vivo* data that support the demonstration *in vitro* that the MukB interaction with TopoIV stimulates catalysis by TopoIV. Finally, we show that MukBEF directs the normal positioning of sister origins after their replication and during their segregation. Overall, the data support models in which the coordinate and sequential action of TopoIV and MukBEF plays an important role during bacterial chromosome segregation.

## INTRODUCTION

Successful propagation of cells relies on the fidelity of chromosome replication and segregation and the processes that compact and organize the chromosome. In bacteria, much is known about the mechanism of replication and its spatial organization, but far less is known about how the chromosome is organized and segregated (reviewed in references 1 and 2). In extensively studied bacteria, segregation of most genetic loci occurs soon after replication, once the precatenanes that hold newly replicated sister loci together have been removed by the type II topoisomerase, topoisomerase IV (TopoIV) (3–6). TopoIV action on precatenated sisters can be modulated by SeqA, which interacts with newly replicated DNA (7). Structural maintenance of chromosome (SMC) complexes, which are present in most organisms, have been implicated in both bacterial chromosome organization and segregation, although their precise functional role and biochemical action remain unclear (reviewed in references 8 and 9).

The *Escherichia coli* SMC complex, MukBEF, was identified through a genetic screen, which initially identified a Muk^−^ mutant that generated anucleate cells and displayed temperature-sensitive growth in rich medium (10, 11). MukB is a 170-kDa protein of distinctive SMC architecture that dimerizes through a dimerization hinge located at one end of an ~50-nm long intramolecular coiled-coil and has an ATPase formed by the N- and C-terminal portions of the protein at the other end of the coiled-coil (9, 12, 13). A kleisin-like protein, MukF, bridges the two ATPase heads in a MukB dimer, while a third protein, MukE, binds to MukF. Loss of function of any of these three proteins leads to the same Muk^−^ phenotype. Functional fluorescent fusions of MukB, MukE, or MukF all associate with the replication origin region (*ori*) of the *E. coli* chromosome in live-cell imaging assays that observe fluorescent-focus formation (14, 15) in reactions that require ATP binding and hydrolysis by MukBEF (16, 17; our unpublished data). In the absence of functional *E. coli* MukBEF, *ori*s are relocated from midcell to an old pole in newborn cells, with the whole chromosome undergoing an apparent 90° rotation within a cell (14).

Biochemical experiments *in vitro* have shown that the MukB hinge interacts with the C-terminal domain of ParC, the catalytic subunit of TopoIV (18–20), leading to the proposal that these proteins collaborate in chromosome disentanglement (20). This interaction stimulates TopoIV-mediated relaxation of negatively supercoiled DNA, but not positively supercoiled DNA *in vitro*. Because negative supercoils have the same right-handed chirality as replicative precatenanes (see Fig. S1 in the supplemental material), we predict that MukBEF should also stimulate decatenation by TopoIV, the prelude to chromosome segregation, raising the possibility that MukBEF and TopoIV act in sequential steps during chromosome segregation. Nevertheless, an *in vitro* study failed to demonstrate MukBEF-stimulated decatenation of multiply linked plasmid replicative catenanes, leading to the proposal that the MukBEF stimulation of TopoIV activity is involved in intramolecular chromosomal events rather than in decatenation (21). The failure of MukB to stimulate decatenation in *in vitro* assays may reflect the fact that the *in vitro* assay conditions failed to recapitulate *in vivo* conditions somehow, such as the absence of MukEF proteins or the fact that *in vivo* proper loading of a complete MukBEF complex onto DNA is required for this stimulation.

The role of TopoIV in decatenation *in vivo* has been widely documented, as has its ability to relax supercoils *in vitro* and *in vivo* (20, 22–25). After TopoIV impairment, DNA replication and reinitiation along with cell growth appear to continue normally, although decatenation of newly replicated loci is blocked as measured by failure to segregate newly replicated sisters (3). Therefore, any action of TopoIV in supercoil removal ahead of a replication fork can be compensated for by the action of other topoisomerases. These observations support the view that the major role of TopoIV *in vivo* is in decatenation rather than supercoil relaxation (3). Indeed, any MukBEF-stimulated negative supercoil relaxation would compound the *in vivo* unlinking problem because it would act to increase the overall linkage between duplex strands in the chromosome. Other studies in both prokaryotes and eukaryotes have implicated functional interactions between SMC complexes and type II topoisomerases and have suggested that these are important for decatenation, although it is not always clear whether these interactions also influence the activity of the topoisomerase in regulating supercoiling, which is important for chromosome organization (5, 26–28).

To address whether MukBEF and TopoIV act in a coordinated manner *in vivo*, we analyzed the functional relationship between MukBEF and TopoIV in live *E. coli* in which a single round of replication was initiated and completed in the same cell cycle. Functional fusions of the fluorescent protein, mYPet, to the ParC and ParE subunits of TopoIV formed foci that associated specifically with fluorescent foci of functional MukBEF, which frequently localized with the *E. coli ori* region. The TopoIV foci were dynamic and did not always form discrete spots, probably because of a high dissociation rate of ParC from MukB, making it necessary for us to use a quantitative cumulative distribution method that assessed the distances between the centroid of Gaussian-fitted MukBEF fluorescent foci and the highest pixel intensity exhibited by fluorescent ParC or ParE, which likely marks the cellular site where the complex exhibits the highest residence time. Using this method, we demonstrated that the two subunits of TopoIV colocalize preferentially with MukBEF foci throughout the cell cycle and also in the absence of replication. Specific depletion experiments additionally showed that MukBEF in foci directs TopoIV to the *ori* region. Additionally, we provide evidence that MukBEF in foci positions *ori* rather than vice versa. Taken together, the data implicate MukBEF as a key component of the DNA segregation process by acting in concert with TopoIV to promote decatenation and positioning of newly replicated *ori*s.

## RESULTS

### Topoisomerase IV associates with MukBEF in live *E. coli*.

**FIG 1:**
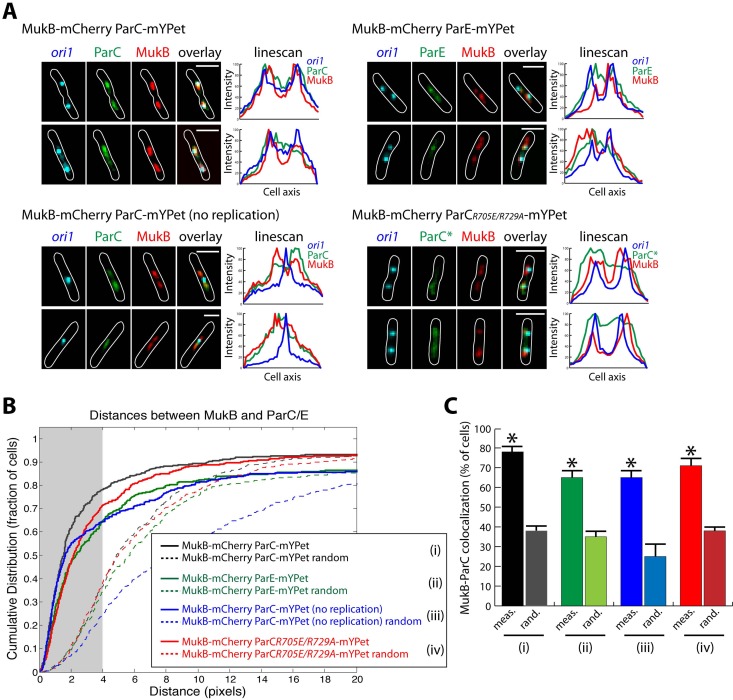
Association of TopoIV subunits ParC and ParE with MukBEF *in vivo*. (A) Representative examples of colocalization events between MukB-mCherry and ParC/E-mYPet proteins within *E. coli* cells. The origin of replication region was identified by the binding of a TetR-CFP protein on a *tetO* array (*ori1*) located 15 kb CCW of the replication origin, *oriC*. The fluorescence profiles of the individual cells show the distribution of fluorescence intensities. Exponential-phase cells were grown in M9-glycerol medium at 30°C prior to imaging. Cells without replication are *dnaC*(Ts) strains grown at the restrictive temperature for 120 min. Bars, 2 µm. (B) In order to localize the fluorescent foci corresponding to MukB and ParC/E proteins or the *ori1* DNA locus within cells, microscopy images were first analyzed using the MicrobeTracker Suite (http://microbetracker.org/) to detect and outline bacterial cells. Cumulative distributions present the distances between the centroids of MukB foci and the brightest ParC/E pixels (Materials and Methods). Colocalization (gray shaded rectangle) is defined as when the MukBEF focus centroid is 4 or less pixels (516 nm) from the brightest ParC/E pixel. (C) The percentages of colocalization between MukBEF and ParC/E were plotted in a histogram. Error bars represent the 95% confidence interval. Asterisks indicate that the measured values (meas.) are statistically significantly different from the random calculated values (rand.) (Materials and Methods).

To address whether we could obtain evidence for an interaction of the ParC and ParE subunits of TopoIV with MukBEF *in vivo*, we replaced the endogenous ParC and ParE genes with functional fluorescent mYPet fusions to ParC and ParE expressed from their endogenous promoters on the *E. coli* chromosome and analyzed mYPet fluorescence in live cells using wide-field epifluorescence imaging. The cells also expressed MukB-mCherry and had their *ori* region marked with fluorescent tetracycline repressor (TetR-CFP) bound to an array of *tet* operators 15 kb counterclockwise (CCW) of *oriC* (*ori1*). Cells with the fluorescent fusions showed near-normal flow cytometry profiles and doubling times (see Fig. S2A and S2C in the supplemental material). Fluorescent MukBEF forms foci that are frequently associated with *ori*, irrespective of whether cells are growing exponentially, or nonreplicating because of a block in replication initiation (Fig. 1A) (14, 15, 17).

Although we observed fluorescent ParC-mYPet (ParC fused to mYPet fluorophore) and ParE-mYPet foci in both steady-state and nonreplicating *dnaC*(Ts) cells (Fig. 1A) (29), they were typically not as well defined as the *ori1* and MukBEF foci, and a high background of ParC/ParE outside foci was evident. This is not surprising, given that *in vitro* analysis has shown a rather weak interaction of ParC with MukB (*K*_*d*_ [dissociation constant] of ~0.5 µM) (18). Initial analysis by simple observation of all three fluorescence channels showed that the ParC-mYPet and ParE-mYPet foci were frequently associated with *ori1* and MukBEF, indicative of an *in vivo* association with MukBEF and/or *ori1* (Fig. 1A, images). Analysis of the three fluorescence profiles showed overlapping peaks for MukBEF, *ori1*, and ParC/E (Fig. 1A, line scans).

In order to establish a quantitative and more objective assessment of the relative localizations of TopoIV and MukBEF, we used automated image analysis that determined the centroid of MukBEF foci by fitting elliptical Gaussian functions. We examined the cumulative distributions of the distances between the brightest ParC/E pixels, which identify the population of ParC/E molecules with the highest residence time and the centroid of the nearest MukBEF focus (Fig. 1B and C; Materials and Methods). For a negative control and to evaluate the level of random coincidence of foci, we performed the same analysis on a simulated random distribution of TopoIV foci within the same cells. The association of TopoIV with MukBEF was now unequivocal: 65% to 78% of the brightest ParC or ParE pixels were located within a distance of 4 pixels (516 nm) from a MukBEF focus, whereas the random distribution yielded 33% and 38%, respectively, for ParE and ParC versus MukBEF. Furthermore, a ParC mutant (ParC with an R-to-E change at position 705 and an R-to-A change at position 729 [ParC_R705E/R729A_]), which showed an impaired interaction with MukB *in vitro* (19), retained almost the same level of colocalization with MukBEF, indicative of at least residual binding to MukBEF *in vivo* (Fig. 1; 72% for mutant ParC compared to 78% for wild-type ParC). Comparable analysis showed similar high levels of colocalization of ParC or ParE with *ori1* (see Fig. S2E in the supplemental material; 78% for ParC, 71% for ParE, with reduced colocalization of ParC_R705E/R729A_ with *ori1* [61%]), as well as MukBEF with *ori1* (Fig. S2D). In our experience, it is not unusual for amino acid substitutions in proteins to lead to loss of *in vitro* activity, but the proteins retain at least some *in vivo* activity.

In nonreplicating *dnaC*(Ts) cells, MukBEF often formed foci close to and on both sides of the single *ori1* focus [Fig. 1A, images in the MukB-mCherry ParC-mYPet (no replication) panel; ~66% of single *ori1* foci were associated with two such MukBEF foci after 120 min at the restrictive temperature]. In this case, ParC was associated with MukBEF rather than with *ori1*, demonstrating that the primary association of TopoIV is with MukBEF rather than *ori1*. The observation that ParE colocalization with MukBEF or *ori1* was always lower than that of ParC with MukBEF/*ori1*, when measured by the cumulative distribution of distances, likely reflects the fact that the ParE association with MukB is via ParC and that there is a significant dissociation rate *in vivo* between the two TopoIV subunits.

We conclude that the robust TopoIV-MukBEF colocalization demonstrated here likely reflects an interaction of MukBEF present in foci with TopoIV *in vivo*, thereby recapitulating the characterized *in vitro* reactions. We are confident that the observed association between ParC/ParE and MukBEF/*ori1* is physiologically relevant and not an artifact for the following reasons. ParC-mYPet and ParE-mYPet fluorescent foci form independently at the same cellular positions where they each colocalize with MukBEF-mCherry in cells that retain MukBEF and TopoIV function. ParC-mYPet and ParE-mYPet fluorescent foci form and colocalize with *ori1* in the absence of labeled MukBEF (but dependent on the presence of functional MukBEF; see Fig. S2F in the supplemental material). Furthermore, the slightly reduced colocalization of the ParC double point mutant that has impaired interaction with the MukB hinge *in vitro* likely relates to the physiological relevance of the association between MukBEF and TopoIV. Finally, extensive analysis of a range of MukB and MukE fusions to mYPet, green fluorescent protein (GFP), and mCherry fluorophores has not only failed to show a fluorophore-dependent interaction but demonstrated *in vivo* localization and function dependent on ATP binding and hydrolysis (16, 17; our unpublished data). Similarly, experiments with the same combinations of fluorophores fused to replisome proteins did not reveal any fluorophore-interaction artifacts (30).

Because of the high background of TopoIV fluorescence, we cannot eliminate the possibility that TopoIV is additionally associated with other regions of the chromosome or with proteins elsewhere; indeed, since many of the MukBEF molecules are not in foci (17), interaction of these molecules with TopoIV could be responsible for at least some of the observed background. Colocalization of ParC foci with the replisome marker, DnaN, was close to random and no higher than that of the association of DnaN with *ori1* (data not shown), as expected from our observation that ParC forms foci in *dnaC*(Ts) cells that are not undergoing replication (Fig. 1A). We are therefore unable to provide evidence that supports the earlier observations that ParC and/or SMC complexes, interact with the bacterial replisome (31–33).

### MukBEF within foci positions TopoIV.

**FIG 2:**
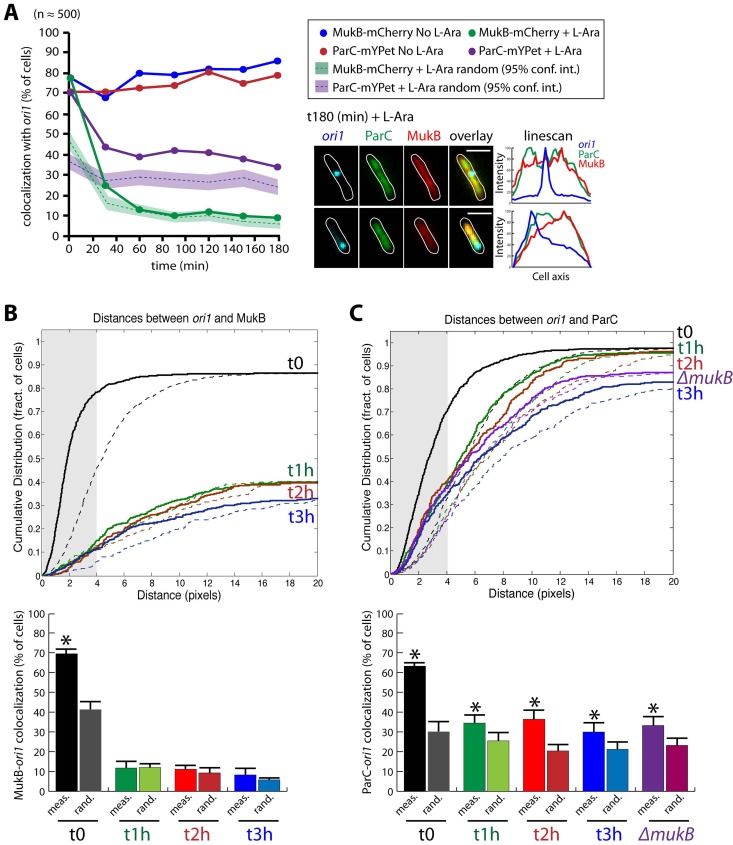
MukB is necessary for TopoIV localization at the origin of replication. (A) Colocalization frequencies (percentages of colocalization determined at a 4-pixel distance) between *ori1* versus MukB and *ori1* versus ParC were recorded during depletion of the MukE protein. Conditions in the absence or presence of l-arabinose (l-Ara) were plotted. The corresponding random curves were also plotted and show that the measured distances are getting closer to the random positioning during the time course of the experiment. Two representative examples of cells after 180 min of MukE depletion are shown. The values in the 95% confidence interval (95% conf. int.) are shown by purple or green shading. Bars, 2 µm. (B) Cumulative curves of the pairwise distances between *ori1* and MukB during the time course of MukE depletion. fract., fraction. (C) Cumulative curves of the pairwise distances between *ori1* and ParC during the time course of MukE depletion. In panels B and C, the percentages of colocalization are plotted in the histograms below the graphs (as in Fig. 1C).

In order to establish whether the observed colocalization between MukBEF and TopoIV is directed by MukBEF, TopoIV, or *ori1*, we used a MukE degron to deplete functional MukBEF (15). Representative cells in which MukE had been depleted are shown in Fig. 2A (images). The MukB-mYPet signal was now dispersed, whereas the *ori1* foci remained intact. All evidence of clear ParC-mYPet focus colocalization with *ori1* had also disappeared, with no clear foci elsewhere. Quantitative analysis showed colocalization between MukBEF, or ParC-mYPet, and *ori1* had almost completely disappeared after 30 min of MukE depletion (Fig. 2A, left). The cumulative distributions showed that the distance between an *ori1* focus and a MukBEF focus or the brightest ParC pixel had become similar to that of the random distribution of localizations after 1 h of MukE depletion and the same as in a *ΔmukB* strain (Fig. 2B and C; see Fig. S3A in the supplemental material). The loss of ParC foci occurred also after MukE degradation in nonreplicating cells, conditions in which cell growth continued (Fig. S3B). In the absence of functional MukBEF, a low level of ParC localization with *ori1* remained (Fig. 2C, bar graph; Fig. S3). This may reflect the fact that action of TopoIV at *ori1* is required for timely *ori1* segregation in the absence of functional MukBEF.

### TopoIV depletion does not abrogate MukBEF foci.

**FIG 3:**
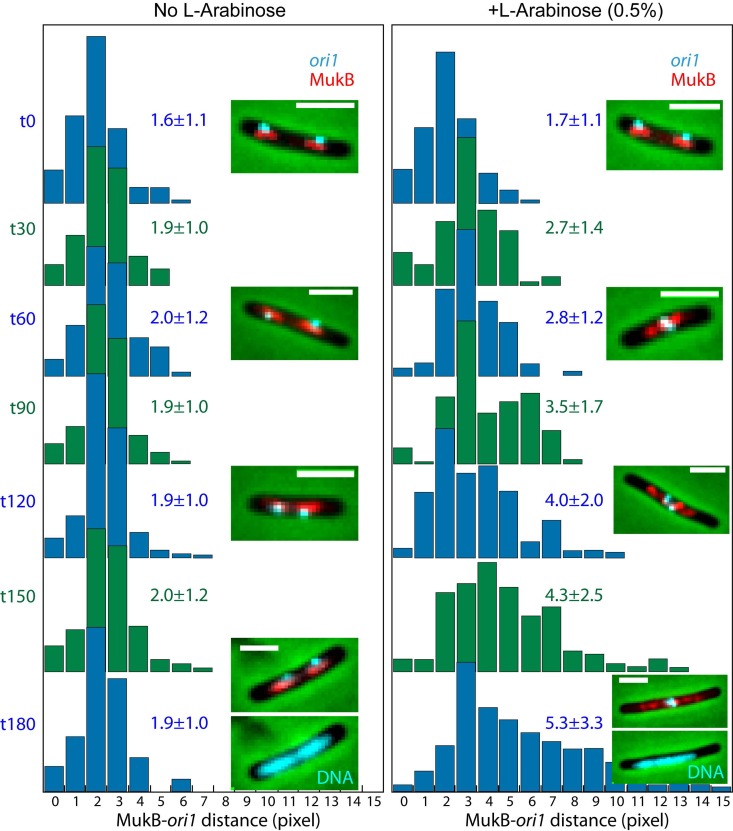
ParC depletion leads to a dispersion of MukB foci. ParC protein was fused to a degron tag. Upon addition of l-arabinose, ParC was efficiently degraded in less than 1 h (see Fig. S4 in the supplemental material). Images were taken at different time points (from 0 to 180 min) after the addition of l-arabinose, and the pairwise distance between MukBEF foci and *ori1* foci was assessed manually using ImageJ software. Means and standard deviations were calculated. Representative examples of cells at each time point are shown. The nucleoid was highlighted by 4′,6′-diamidino-2-phenylindole (DAPI) staining for the examples at t180 (180 min). Bars, 2 µm.

When the complementary experiment was undertaken, in which a degron derivative of ParC was depleted efficiently (see Fig. S4A in the supplemental material), MukBEF foci persisted. By 1 h of depletion, most cells had a single *ori1* focus or two closely spaced *ori1* foci, consistent with the expected impairment in decatenation of the *ori* region (Fig. 3, compare left and right panels) (3). In most cells, several MukBEF foci were evident, evenly spaced throughout the nucleoid and placed on both sides of *ori1*, sometimes with a high background throughout the nucleoid (e.g., see Fig. 3, 120 min with l-Ara). Two to four MukBEF foci were present in most cells, a number similar to the number of *ori1*s expected in such cells given that replication continues despite the inhibition of chromosome segregation and cell division after TopoIV impairment (3). The apparent regular position of the MukBEF foci within the nucleoid corresponded approximately to where *ori1*s would have been if their decatenation and segregation had been possible.

When a similar ParC depletion experiment was repeated in the absence of DNA replication, using a *dnaC*(Ts) allele at the restrictive temperature, the MukBEF foci persisted, with the majority of cells containing MukBEF foci close to and on either side of the single *ori1* focus, as in ParC^+^ cells (see Fig. S4B in the supplemental material). This confirms that MukBEF foci persist in the absence of TopoIV and that the dispersed multiple MukBEF foci observed in replicating cells are dependent on ongoing DNA replication and/or the consequent accumulation of chromosomal DNA.

**FIG 4:**
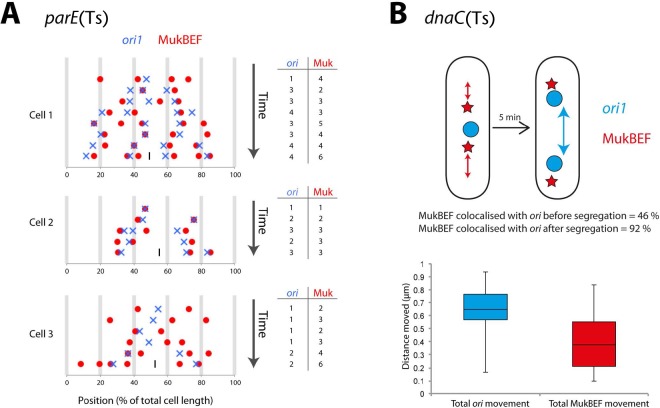
Regular spacing of *ori*-independent MukBEF foci after TopoIV impairment and after inhibition of replication initiation. (A) Summary of the time-lapse analysis for 3 representative cells released from a *parE*(Ts) arrest. Blue × symbols represent origin peaks, and red circles represent MukBEF peaks in the line profiles of intensity along the cells. Time moves down the *y* axis, and the time points are as follows: 10, 30, 60, 90, 120, 150, 180, and 210 min after release from arrest. For each cell, the analysis is shown up until the point where the cells had clearly divided; septa are marked by a vertical black line on the graph at the last time point. The tables to the right of the three graphs show the number of *ori* and MukBEF peaks at each time point. The example cells represent 3 cell types observed after *parE* arrest—cells where the number of Muk foci are equal to (or less than double), double or more than double the number of *ori* foci—these types are equally represented within the population. (B) Time-lapse following *ori1* and MukBEF foci after release from *dnaC*(Ts) arrest. We measured the total distance moved by *ori1* and MukBEF foci along the long axis of the cell during the 5-min interval spanning origin segregation, which is illustrated in the top panel (the blue arrow indicates total distance moved by *ori*, and the two red arrows combined are the total distance moved by Muk). The distances measured are shown in the box plot below the schematic drawing of the cell; the box indicates the interquartile range with the central line of the box indicating the median value. The whiskers of the plot indicate the minimum and maximum measurements. Colocalization was assessed manually, and it was defined as when the foci centers were less than 2 pixels (258 nm) apart.

When ParE activity was impaired using a thermosensitive mutation at the restrictive temperature, a similar behavior was observed after 120 min at the restrictive temperature: cells contained a single *ori1* focus or closely spaced *ori1* foci, with ~4 evenly spaced MukBEF foci throughout the nucleoid (see Fig. S4C in the supplemental material). On return to the permissive temperature, a proportion (~30%) of cells showed a similar distribution of MukBEF foci, but now with *ori*s colocalized. The remaining fraction of cells seemed unable to decatenate and segregate their sister *ori1*s. To characterize the changes in distribution of MukBEF and *ori1* foci during the transition from TopoIV-impaired to functional TopoIV, we undertook time-lapse experiments using *parE*(Ts) cells in which *ori1* and MukBEF were differentially fluorescently labeled (Fig. 4 and Fig. S5). As expected, the temperature shift to 42°C resulted in cells containing either a single *ori1* focus close to midcell, or two separated *ori1* foci at approximately cell quarter positions. Given that cells had been maintained for just over 1 cell generation at 42°C prior to the shift back to the permissive temperature, we expected each *ori1* focus to contain 2 to 4 catenated *ori1* regions at shift-down. Again we observed ~30% of cells went on to segregate their *ori1* regions and to eventually divide during the 5-h time-lapse experiment at the permissive temperature; in general, these were the shorter cells. In order to simplify the analysis, we focused on cells that started with a single *ori1* focus, although the cells starting with two separated *ori1* foci showed comparable behavior. We also analyzed only cells that went on to divide into normal looking and growing cells. Typical behavior is shown in the schematic in Fig. 4A, with the primary data for the same three cells shown in Fig. S5. During the 300-min time-lapse experiment, the initial single centrally placed *ori1* focus segregated to give 2 to 4 well-separated *ori1* foci, with the initial segregation event occurring anywhere between 30 min and 120 min after the shift to the permissive temperature. By the end of the experiment, the segregated *ori1* foci were now frequently positioned coincident with or close to a MukBEF focus. We noted that the positions of MukBEF foci before the shift to the permissive temperature were similar to the positions they take up at times immediately prior to cell division. It is tempting to speculate that the MukBEF foci had taken up their final positions before the origins were able to segregate and that this then led to *ori* positioning; however, this observation is complicated by the fact that the MukBEF foci themselves are mobile during the time-lapse.

### MukBEF foci position sister *ori* regions when the TopoIV-MukBEF association is retained.

In the experiments that exploited TopoIV impairment and in those using TopoIV-positive (TopoIV^+^) *dnaC*(Ts) cells, we observed that the association of MukBEF foci with *ori1* was reduced, with MukBEF foci taking up regular positions on both sides of *ori1*. These observations led us to consider that MukBEF focus formation is independent of *ori* although dependent on the nucleoid, because MukBEF foci always formed on nucleoids rather than in the nucleoid-free space (14). In order to explore the relationship between *ori*s and MukBEF foci, we performed time-lapse experiments with a synchronized TopoIV^+^ *dnaC*(Ts) strain released after 105 min at the restrictive temperature, conditions under which we know that the majority of cells have a single unreplicated *ori* and no associated replisome (29). The cells expressed MukE-mYPet and had their *ori* region marked with fluorescent Lac repressor (LacI-mCherry) bound to an array of *lac* operators 15 kb CCW of *oriC* (*ori1*). The strain was released into permissive conditions to allow initiation of replication, and *ori1* and MukBEF foci were monitored using time-lapse microscopy. We focused on two time points 5 min apart that span the interval in which the replication origin foci segregate (Fig. 4B, top portion). We observed that *ori1* is generally colocalized with a MukBEF focus both before and after *ori1* segregation; however, there are multiple MukBEF foci at times when there is only a single *ori1* focus and not all MukBEF foci are colocalized with *ori1*. We observed that once the *ori1* foci have segregated, the colocalization of MukBEF foci with *ori1* foci was increased; before *ori1* segregation, 46% of the MukBEF foci colocalized with *ori1*, whereas after *ori1* segregation, 92% of MukBEF foci colocalized with *ori1* (colocalization was defined as when the foci centers were less than 2 pixels [258 nm] apart). We also measured the total distances these foci moved along the long axis of the cell during the 5-min interval spanning *ori1* segregation. As shown in Fig. 4B, we found that *ori1* foci moved a greater total distance (median, 0.65 µm) than the distance moved by the MukBEF foci (median, 0.38 µm). This greater movement of the *ori1* foci and the increased localization of MukBEF foci with *ori* after segregation support the idea that the *ori* region moves to the position of the MukBEF foci, and therefore, that MukBEF foci play a role in positioning segregated origin regions.

## DISCUSSION

We have demonstrated that MukBEF and TopoIV associate *in vivo* and that this association occurs in the absence of DNA replication and at all stages of the cell cycle. We think it is highly likely that this association reflects the ParC-MukB interaction demonstrated *in vitro* (18, 19, 21). A key unanswered question regards the functional significance of the *in vivo* TopoIV-MukBEF association demonstrated here; specifically, does the MukBEF-directed association of TopoIV with *ori* modulate sister cohesion in the *ori* region by stimulating decatenation?

Consistent with this hypothesis, earlier work and the work here have demonstrated that in Muk^−^ cells, newly replicated *ori1*s can exhibit delayed segregation (14) (see Fig. S6 in the supplemental material), suggesting the possibility that the recruitment by MukBEF of TopoIV to the *ori* region facilitates decatenation of newly replicated sister *ori*s. A quantitative demonstration of this effect was shown when we used a degron to deplete MukE, thereby leading to an abrogation of MukBEF function; the fraction of steady-state cells containing a single *ori1* focus increased to 64% from 25% after MukBEF impairment (Fig. S6A). In order to test the hypothesis directly, we analyzed *ori1* segregation in the ParC_R705E/R729A_ mutant that is impaired in its interaction with MukBEF *in vitro*, although any *in vivo* impairment appears to be modest. Snapshot analysis of steady-state cells showed an increase in the fraction of single *ori1* focus cells from 26% to 37% in the mutant compared to the wild type, while time-lapse analysis showed an increase in cohesion time from 18 min to 23 min in the mutant compared to the wild type, when the time of *ori1* segregation after replisome appearance was measured (Fig. S6A and S6B). These apparent modest defects in decatenation are consistent with, but do not prove, a model in which the TopoIV-MukBEF interaction directs TopoIV-mediated decatenation to *ori*. A ParC mutant that is catenation proficient but is totally defective in the *in vivo* interaction with MukBEF (or the complementary mutant for MukB) would be needed to test the hypothesis directly.

The demonstration *in vitro* that the relaxation activity of TopoIV on right-handed (negative) supercoiled DNA, but not on left-handed (positive) supercoiled DNA, is stimulated by the interaction with MukBEF (21) suggests that the interaction should enhance decatenation because negative supercoils have the same right-handed chirality as replicative catenanes (see Fig. S1 in the supplemental material). Taken together, the *in vitro* stimulation of TopoIV activity on a substrate that mimics replicative catenanes and the observation of an *in vivo* association of MukBEF and TopoIV with *ori* makes us confident that TopoIV will be directed to *ori* to remove catenanes from newly replicated sisters. TopoIV may also be associated with the majority of MukBEF molecules that are not present as foci, therefore allowing TopoIV to act globally on the chromosome.

Our observations also lead us to conclude that MukBEF complexes within foci act to position the *ori* region and to additionally direct a fraction of TopoIV molecules to *ori*. Ablation of MukBEF leads both to *ori* mispositioning (14) and to the loss of ParC foci (this paper). Our observation that TopoIV impairment did not prevent MukBEF focus formation makes us confident that it is the MukBEF within foci that positions TopoIV, a conclusion supported by our observation that in *dnaC*(Ts) cells grown at 37°C, MukBEF foci on either side of *ori1* colocalize with ParC rather than *ori* itself. We do not know what is positioning the MukBEF foci, but the regular patterning is reminiscent of that which occurs when ParAB-*parS* systems position low-copy plasmids, chemosensory apparatus, or carboxysomes on the nucleoid (1, 34). Since *ori* positioning and chromosome segregation are disrupted by Muk impairment, the data provide strong support for a model in which the coordinated and sequential action of TopoIV and MukBEF in complexes visualized as foci plays a central role in initiating segregation of newly replicated *ori*s.

An earlier study of TopoIV in *E. coli* found that the catalytic subunit, ParC, was associated with the replisome, while the other subunit, ParE, was located in nucleoid-free regions of the cell (31). Based on this and other observations, it was proposed that TopoIV activity is regulated temporally, with activity occurring at late stages of the cell cycle when ParC could be released from the replisome and associate with the FtsK translocase (35). We do not know how to reconcile this information with the results obtained in this work and in our earlier work (3), which together show that TopoIV can act at *ori* through its association with MukBEF. It is difficult to imagine how a single replisome, or FtsK complex, could interact with a substantial proportion of cellular TopoIV. Biochemical characterization has also shown that ParCE complexes are stable during gel filtration and ultracentrifugation (22), and therefore, it seems likely that a substantial proportion of ParC and ParE in cells can form functional topoisomerase molecules. We note that in the cell biology analysis of reference 31, immunocytochemistry was used in fixed rapidly growing cells, a situation where resolution is not at its highest. Finally, the roles of TopoIV in decatenating newly replicated regions of the chromosome and in replicating plasmids, which are positioned in the extranuclear space, have been documented (3, 7, 22, 36). A genetic study of *Bacillus subtilis* indicated an interaction between the SMC complex and TopoIV (26), while studies of the budding yeast *Saccharomyces cerevisiae* have implicated functional interactions of both cohesin and condensin with topoisomerase II that are important in chromosome decatenation (27, 28, 37, 38). It therefore seems possible that functional interactions between SMC complexes and topoisomerases are ubiquitous.

## MATERIALS AND METHODS

### Bacterial strains and growth.

The bacterial strains used in this study are listed in Table 1. The plasmids and oligonucleotides used in this study are shown in Table S1 in the supplemental material. All strains are derivatives of the *Escherichia coli* K-12 AB1157 strain (39). Fusion of genes with fluorescent or degron tags was performed using the λRed method (40). Fused genes were transferred to generate the final strains through P1 phage transduction (41). For multiple insertions of modified genes, the Kan^r^ gene was removed using site-specific recombination through expression of the Flp recombinase from the pCP20 plasmid (40). All strains were constructed and analyzed several times independently in order to avoid any effect of potential suppressor mutations. *dnaC*(Ts) cells were grown at 37°C for 120 min to generate a population of cells that could not reinitiate replication, as described previously (15, 29). Depletion of proteins carrying a degron tag necessitates the presence of the SspB protein which is expressed chromosomally under the arabinose-controlled promoter (pAra) in order to control the timing of the depletion (15). *tetO* and *lacO* arrays (240 copies) are inserted at 15 kb counterclockwise (CCW) of *oriC* (*ori1*).

**TABLE 1:** Bacterial strains used in this study^*a*^

Strain	Genotype^*b*^
AU2019	*lacO* at *ori1 hyg*; *tetO* at *ter3 gen*; *plac*-*lacI*-*mCherry* at *leuB*; *plac*-*lacI*-*tetR*-*mCerulean* at *galK*; *mukE*-*mYPet dnaC2 thrA*::Tn*10*
ENOX5.130	*mukB*::*mCherry frt*; *parC*::*mYPet kan*; *plac-tetR*::*cfp frt*; *tetO* at *ori1 gen*
ENOX5.138	*mukB*::*mCherry frt*; *parE*::*mYPet frt*; *plac-tetR*::*cfp kan*; *tetO* at *ori1 gen*
ENOX5.147	*mukB*::*mCherry frt*; *parC*::*mYPet kan*; *plac-tetR*::*cfp frt*; *tetO* at *ori1 gen*; *dnaC2*(Ts) *thrA*::Tn*10*
ENOX5.225	*mukB*::*mCherry frt*; *parCR705E/R729A*::*mYPet kan*; *plac-tetR*::*cfp frt*; *tetO* at *ori1 gen*
ENOX5.167	*mukE*::*degron frt*; *mukB*::*mCherry kan*; *parC*::*mYPet frt*; *plac-tetR*::*cfp frt*; *tetO* at *ori1 gen*; *ΔsspB frt*; *PAra-sspB frt*
ENOX5.182	*ΔmukB kan*; *parC*::*mYPet frt*; *plac-tetR*::*cfp frt*; *tetO* at *ori1 gen*
ENOX5.47	*parC*::*degron frt*; *mukB*::*mCherry frt*; *plac-tetR*::*cfp frt*; *tetO* at *ori1 gen*; *ΔsspB frt*; *PAra-sspB frt*
ENOX5.41	*parC*::*degron frt*; *mukB*::*mCherry frt*; *plac-tetR*::*cfp kan*; *tetO* at *ori1 gen*; *ΔsspB frt*; *PAra-sspB frt*; *dnaC2*(Ts) *thrA*::Tn*10*
ENOX5.56	*parE*(Ts); *mukB*::*mCherry frt*; *plac-tetR*::*cfp kan*; *tetO* at *ori1 gen*
ENOX5.245	*frt mCherry*::*dnaN*; *parC*::*mYPet kan*; *plac-tetR*::*cfp frt*; *tetO* at *ori1 gen*

^a^ All these strains were constructed in this study.

^b^ Abbreviations: *kan*, kanamycin resistance gene; *hyg*, hygromycin B resistance gene; *gen*, gentamicin resistance gene; *frt*, FLP site-specific recombination site.

### Microscopy.

Image captures were performed as described in reference 15. Cells were grown at 30°C in M9 medium supplemented with appropriate amino acids and 0.2% glycerol (M9-gly). For experiments in the absence of replication, strains carrying the *dnaC*(Ts) allele were shifted 2 h at 37°C (to synchronize cells by allowing completion of existing rounds of replication but preventing any further replication initiation), and the microscope chamber was maintained at 37°C in order to prevent any reinitiation of replication during image acquisition. Induction of SspB protein during depletion experiments was done by addition of 0.5% l-arabinose. For microscopy, cells in exponential phase (*A*_600_ ≈ 0.1) were concentrated and laid on a 1% M9-gly agarose pad on a slide. During depletion experiments, the l-arabinose was also maintained in the slides when required.

For the *parE*(Ts) time-lapse experiments, cells were grown at 30°C in M9-gly to an *A*_600_ of ~0.05. The cells were shifted to 42°C for 2 h, and then a sample was spun down and spotted onto a prewarmed 1% M9-gly agarose pad on a slide. The cells were imaged at 30°C for 5 h; the initial images were taken 10 and 30 min after release and then every 30 min up until 300 min.

For the *dnaC*(Ts) time-lapse experiments (Fig. 4B), cells were grown at 30°C in M9-gly to an *A*_600_ of ~0.05. The cells were shifted to 37°C for 105 min (the restrictive temperature for *dnaC2*). A sample was spotted onto a prewarmed 1% M9-gly agarose pad on a slide. The cells were released to the permissive temperature of 30°C to allow replication initiation and imaged every 5 min for 2 h.

### Image analysis.

Images were taken and processed by Metamorph 6.2, and image analysis was done using ImageJ or specific programs run in Matlab.

### (i) Semiquantitative analysis of fluorescence distributions.

Fluorescence distributions within cells were plotted as line scans using the Plot Profile command of ImageJ or MicrobeTracker software run in Matlab (42). Maximum intensity values were normalized between 0 and 100% for each channel before plotting. For the *parE*(Ts) experiments (Fig. 4A), peaks in intensity were defined as peaks when the height of the peak was 5% or more of an increase in intensity above the neighboring points of inflection.

### (ii) Quantitative analysis of colocalization.

Cell outlines were first delineated from a phase image using the MicrobeTracker software run in Matlab (see Fig. S7 in the supplemental material). This segmenting analysis created a “mesh” for each cell, within which each pixel is characterized by a specific *x*,*y* coordinate (Fig. S7Bi). This step is critical to determine pairwise distances between two independent pixels of different channels within a specific cell (see further steps).

The second step of the analysis was to find the population of fluorescent molecules with the highest residence time within a cell. These populations of molecules assembled as local maxima of fluorescence intensity. As both TetR-CFP (cyan fluorescent protein) bound to the *ori1* locus and MukB-mCherry formed well-defined foci, we adapted the automated localization analysis method of Holden et al. (43) that first identified candidate foci above an intensity threshold and subsequently determined their centroids by fitting an elliptical Gaussian function (see Fig. S7Biii and S7Biv in the supplemental material). This kind of analysis was not possible for ParC/E-mYPet signal, as ParC/E did not always assemble discrete foci. In order to localize the population of ParC/E molecules with the highest residence time, we determined the brightest pixel within each cell (Fig. S7Bii). It should be noted that the Gaussian localization analysis for MukBEF and *ori1* can identify multiple fluorescent foci within one cell or none at all, but the brightest pixel analysis finds exactly one pixel with the highest intensity for ParC/E.

The third step of the analysis measured the pairwise distances between the brightest ParC/E pixel and the nearest MukBEF or *ori1* localization (see Fig. S7C in the supplemental material). To determine the distribution of distances expected from an entirely random localization of ParC/E, we also calculated distances between a pixel randomly positioned within the cell and the nearest MukBEF or *ori1* focus (Fig. S7C). For MukBEF-*ori1* colocalization analysis with multiple MukBEF foci within each cell, an equal number of random MukBEF localizations was generated per cell, and the smallest pairwise distance was calculated.

In the fourth step, the distances were plotted as cumulative distributions (see Fig. S7D in the supplemental material). A threshold of 4 pixels (516 nm) was chosen to define colocalization. The fraction of cells with colocalizing foci was thus determined from the cumulative distributions at 4-pixel distance with 95% statistical confidence bounds (Fig. S7D). Note that the cumulative distribution curves do not reach 100% of cells even for large distances, because MukBEF or *ori1* foci can be absent in some cells; the asymptotic maximum values hence give the fraction of cells that showed MukBEF or *ori1* foci. For example, ~90% of wild-type cells displayed MukBEF foci (Fig. 1B), which reduced to ~30% MukBEF foci under MukE degron conditions (Fig. 2B).

### (iii) MukB foci distribution during ParC/E depletion.

Because the automated analysis was not sufficiently robust to identify and localize the larger number of closely spaced MukBEF foci during the course of ParC/E depletion, the distances in pixels between centroids of MukB-assembled foci and the *ori1* locus were manually measured using ImageJ software and plotted as histograms.

## SUPPLEMENTAL MATERIAL

Text S1 Further details of Materials and Methods. Download Text S1, DOCX file, 0.1 MB

Figure S1 Topology of replicative (pre)catenanes. Duplex DNA has the two strands of the double helix in a right-handed (RH) (+) configuration (bottom panel, right). Precatenanes that interlink the two sister duplexes after replication have the same RH (+) topology (bottom panel). RH (−) supercoils (SCs) have the same RH chirality as (+) precatenanes, whereas the left-handed (LH) (+) SCs that accumulate ahead of a replication fork have the opposite chirality (bottom left panel). Note that one converts an RH (−) SC to an RH (+) replicative catenane by “cutting” the top and bottom of the plectoneme and rejoining (top panel); the sign changes from (−) to (+) because of the relative change in orientation of the red arrows. Hence, conditions that favor the stimulation of RH (−) SC relaxation are expected to stimulate decatenation, since the substrates have the same RH chirality. Download Figure S1, PDF file, 0.2 MB

Figure S2 Cell cycle parameters of fluorescent fusions and the demonstration that TopoIV subunits colocalize with MukBEF foci and *ori1*. (A) Western blot analysis showing the expression of the full-length versions of the different fusion proteins. (B) Generation times of the strains expressing the different fusion proteins. Cells were grown in LB medium at 30°C. (C) Flow cytometry analysis of cells grown in M9-glycerol medium at 30°C. Exponential-phase cells were analyzed. Strains expressing various versions of ParC/E-fluorescent protein fusions were compared to the AB1157 strain. The table shows the different proportions of 1n, 2n, and >2n content (1n includes chromosomes up to ~50% replicated, while 2n includes chromosomes that are >50% replicated). (D and E) Colocalization events between *ori1* versus MukB (D) and *ori1* versus ParC/E (E). Cumulative curves and histograms were generated as described in the legend to Fig. 1. In panels D and E, black and gray bars correspond to the MukB-mCherry ParC-mYPet *ori1-tetO* (TetR-Cfp) strain (i), green bars correspond to the MukB-mCherry ParE-mYPet *ori1-tetO* (TetR-Cfp) strain (ii), blue bars correspond to the MukB-mCherry ParC-mYPet *ori1-tetO* (TetR-Cfp) strain in the absence of replication (iii), and red bars correspond to the MukB-mCherry ParC*R705E/R729A*-mYPet *ori1-tetO* (TetR-Cfp) strain (iv). (F) Representative examples of cells expressing ParC-mYPet or ParE-mYPet in strains where the origin of replication locus was identified by the binding of a TetR-CFP protein on a *tetO* array (*ori1*). Bars, 2 µm. Download Figure S2, PDF file, 1.5 MB

Figure S3 Deletion of MukB or depletion of MukE leads to loss of ParC colocalization with the origin of replication. (A) Representative examples of cells expressing a ParC-mYPet fusion, with *ori1* labeled in a *ΔmukB* strain. Cells were grown at 22°C in M9-glycerol medium. (B) MukE depletion was performed in the absence of replication [*dnaC*(Ts) allele at 37°C; depletion started after 2 h at 37°C]. Representative examples of cells at different time points after the addition of l-arabinose (0.5%) are shown. The average sizes and standard deviations of 50 independent cells at the different time points are also indicated. Bars, 2 µm. Download Figure S3, PDF file, 0.6 MB

Figure S4 MukB foci persist in *E. coli* cells upon impairment of topoisomerase IV. (A) Western blot showing the disappearance of ParC protein upon the addition of l-arabinose (0.5%) in cultures of *E. coli* in LB or M9 medium. (B) The histograms show the distribution of MukB-*ori1* distances upon depletion of ParC protein in the absence of replication [*dnaC*(Ts) allele at 37°C; depletion started after 2 h at 37°C]. (C) Impairment of ParE activity was achieved by using a thermosensitive allele of ParE [*parE*(Ts)] and shift of growth cultures to 42°C. Return to functional ParE was assayed after shift of the cultures to 30°C. Aliquots were taken at different time points, and the distances (in pixels) between MukB foci and the *ori1* locus was assessed manually using ImageJ software. Means and standard deviations were calculated. Representative examples of cells at each time point are represented. Bars, 2 µm. Download Figure S4, PDF file, 0.6 MB

Figure S5 Time-lapse microscopy following *ori1* and MukBEF dynamics during the transition from impaired TopoIV to functional TopoIV. Line profile analysis of time-lapse microscopic images for 3 representative cells released from *parE*(Ts) impairment at 42°C. These are the same 3 cells summarized in Fig. 4. The blue lines represent intensity profiles for *ori1*, and the red lines represent MukBEF. Cell images are also shown for cell 1. For each cell, the analysis is shown up until the point where the cells had clearly divided; septa are marked by a vertical black line on the graph for the last time point. Download Figure S5, PDF file, 0.5 MB

Figure S6 The TopoIV-MukBEF interaction may direct TopoIV-mediated decatenation to *ori*. (A) Percentage of cells containing a single *ori1* locus in indicated strains. (B) Cumulative distributions describing *ori1* locus segregation time after replisome appearance (mCherry-DnaN) in strains expressing ParC-mYPet or ParC_R705E/R729A_-mYPet fusion. Representative examples of such segregation events are shown. Bars, 2 µm. Download Figure S6, PDF file, 0.6 MB

Figure S7 Analysis of colocalization events. (A) Four different channels were typically imaged during a microscopy experiment: (i) a phase-contrast image, (ii) a fluorescence image in the green channel (GFP filter set) to visualize the mYPet fusion proteins, (iii) a fluorescence image in the blue channel (CFP filter set) to visualize the CFP fusion proteins, and (iv) a fluorescence image in the red channel (mCherry filter set) to visualize the mCherry fusion proteins. (B) Outlines of cells were defined using MicrobeTracker from the phase-contrast image (i), and the generated meshes were used in order to find the brightest pixel by cell in the green channel (ii). Foci assembled by MukB-mCherry (iv) or TetR-CFP (iii) were automatically found using a Gaussian fitting algorithm. (C) Pairwise distances between the closest brightest ParC pixel and the centroids of Gaussian-fitted MukBEF or TetR fluorescent foci were measured (yellow rectangles). Distances between a reference pixel (i.e., from MukB or *ori1* focus) and a randomly positioned pixel in the cell were also calculated. (D) The distances were plotted as cumulative distribution curves. Colocalization frequencies were defined as the proportion of cells showing pairwise distances shorter than 4 pixels. Download Figure S7, PDF file, 2.4 MB

Table S1 Plasmids and oligonucleotides used in this study. Table S1, DOCX file, 0.1 MB.
