# Supplementary material for: The SMC Complex MukBEF Recruits Topoisomerase IV to the Origin of Replication Region in Live Escherichia coli
Source: mBio. 2014 Feb 11;5(1):e01001-13. doi: 10.1128/mBio.01001-13 (PMC3950513; doi:10.1128/mBio.01001-13)
Supplement: Text S1 — Further details of Materials and Methods. Download [file mbo001141739s1.docx]

**Supplemental Information**

**The SMC complex MukBEF recruits Topoisomerase IV to the origin of replication region in live *Escherichia coli***

Nicolas Emilien^1^, Amy L. Upton, Stephan Uphoff, Olivia Henry, Anjana Badrinarayanan^2^ and David Sherratt^,^*

Department of Biochemistry, University of Oxford, Oxford OX1 3QU

*to whom correspondence should be addressed ([david.sherratt@bioch.ox.ac.uk](mailto:david.sherratt@bioch.ox.ac.uk))

Present address:

^1^ Laboratoire du Métabolisme de l’ARN FRS/FNRS-IBMM-CMMI-Université Libre de Bruxelles. Rue Profs Jeener & Brachet, 12, B-6041 Charleroi-Gosselies, Belgium

^2^ Department of Biology, Massachusetts Institute of Technology, 77 Massachusetts Ave., Cambridge, MA 02139, USA

Running head: Topoisomerase IV and MukBEF interact *in vivo*

Keywords: MukBEF, SMC complex, *Escherichia coli*, TopoisomeraseIV, chromosome segregation

**Supplementary Material**

***Construction of chromosomal -mCherry, -mYPet and -degron fusions***

Plasmids and oligonucleotides used in this study are listed in Table S1. New strains were constructed using λRed recombination (1). Oligonucleotides had a 50 nt complementary sequence to the last 50 bp of the gene (not including the stop codon) or 50 bp downstream, followed by 20 nt complementary to a 11 aa linker or the end of a kanamycin resistance cassette in the plasmid, respectively. These oligonucleotides were used in PCR reactions on plasmids carrying the 11 aa linker followed by the sequence coding for the fluorescent proteins mYPet/mCherry or the degron tag and a kanamycin resistance cassette (Table S1). The construction of these plasmids were described previously (2-4). The DNA fragment was gel purified and 1 ug was electro-transformed into AB1157 derivative cells overexpressing λRed proteins from pKD46 (1). Cells were selected on kanamycin plates and insertions into the chromosome were checked by PCR and DNA sequencing. For multiple colour experiments, first λRed fusions were performed and the kanamycin resistance cassette was then removed by the Flp recombinase acting on the FRT sites flanking the resistance gene (1).

For the construction of the ParC mutant carrying the R705E and R729A mutations, we first cloned the ParC-mYPet Kan^r^ cassette (amplified from the chromosome using *ParC-mYPet kan cloning forward* and *reverse* oligonucleotides) within a pUC18-derived vector. Afterwards, a first round of mutagenesis was performed by the QuickChange method (Agilent) using the oligonucleotides *ParC R705E forward* and *reverse* to introduce the R705E mutation. A second round of mutagenesis was then performed using the oligonucleotides *ParC R729A forward* and *reverse* to introduce the second mutation R729A. The all cassette was then re-amplified (using the oligonucleotides *ParC mutant integration forward* and *reverse*) and introduced by λRed into AB1157.

MukE degron construction and the chromosomal integration of the *pAra-sspB* cassette were described in a previous study (4). The design and chromosomal integration of the *plac-tetR-cfp* cassette and *tetO* array at *ori1* site were also described in previous studies (2, 5).

***Western-blots***

Western-blot experiments were carried out in order to assess the expression of the different fusion proteins (Fig. S2) and to check the time course of protein degradation in ParC depletion experiments (Fig. S4A). Western-blot showing MukE depletion (strain ENOX5.167) was described previously (4).

Cells from exponential phase were pelleted and resuspended in MilliQ water according to the A_600nm_ of each sample in order to harmonize the quantity of loaded proteins. An equal volume of 2x protein gel loading buffer (Nalgen) was added and samples were boiled during 10 min. Proteins samples were separated on 6% SDS-PAGE gels during 120 min at 100 Volts, RT°. Proteins were then transferred on nitrocellulose membranes using the iBlot dry blotting system (Invitrogen). Membranes were incubated 1 h at RT° in the blocking solution (TBST + 3% non fat milk) and washed 3 x 5 min in the washing buffer (TBST + 0.5% non fat milk). Then, the membranes were incubated O/N 4°C with the primary antibody (1:1000 in washing buffer). To detect mYPet fluorescent protein, we used the anti-GFP monoclonal antibody JL-8 (Clontech). To detect mCherry fluorescent protein, we used the anti-mCherry monoclonal antibody (Abcam). Proteins fused to a degron tag were also fused in tandem to a myc epitope, which was detected using an anti-myc monoclonal antibody (Sigma). Membranes were washed 3 x 5 min in the washing buffer before the incubation with the secondary antibody (1:10,000 in washing buffer; anti-mouse IgG peroxidase from Sigma) during 1 h at RT°. Membranes were washed again 3 x 5 min in the washing buffer and they were then revealed using the Supersignal WestPico chemiluminescent substrate (ThermoScientific) and imaged using BioRad ChemiDoc.

***Microscopy***

For depletion of the ParE protein snaphot experiments, the strain carrying the *parE^ts^* allele (Table 1) was shifted to 42°C and samples were recovered at the indicated time points. The microscope was also maintained at 42°C to avoid any production of stable ParE protein.

*ΔmukB* cells expressing ParC-mYPet (Table 1) were grown at 22°C before imaging at RT°.

MukE depletion experiment in the absence of replication were performed in a strain carrying a *dnaC^ts^* allele at 37°C to avoid any productive round of DNA replication. Depletion was started by the addition of 0.5% of L-Arabinose 2 hours after the shift at 37°C to allow the completion of ongoing replication before starting the experiment. Samples were analysed at 0, 1, 2 and 3h after the addition of L-Arabinose.

Time-lapse microscopy experiments to determine the *ori1* locus segregation time after replisome appearance were performed at 30°C in M9-glycerol agarose pad with the strain ENOX5.245 (Table 1). Images were captured every 5 min.

***Flow cytometry***

Cells were grown in M9-gly to exponential phase (A_600nm_ ≈ 0.2). 200 μl of the cultures were fixed with 3.5 ml of ice-cold 74% ethanol. For the staining procedure, the cells were pelleted and washed twice in 100 μl of cold staining buffer (10 mM Tris pH 7.4, 10 mM MgCl_2_). The 100 μl samples were mixed with an equal volume of the staining solution Syto-16 (3 μM). 100,000 events were recorded in a Becton Disckinson FACScalibur machine using FL1-H and results were analysed using Weasel.

**Supplemental References**

1. **Datsenko KA**, **Wanner BL**. 2000. One-step inactivation of chromosomal genes in Escherichia coli K-12 using PCR products. Proc Natl Acad Sci USA **97**:6640–6645.

2. **Reyes-Lamothe R**, **Possoz C**, **Danilova O**, **Sherratt DJ**. 2008. Independent positioning and action of Escherichia coli replisomes in live cells. Cell **133**:90–102.

3. **Badrinarayanan A**, **Reyes-Lamothe R**, **Uphoff S**, **Leake MC**, **Sherratt DJ**. 2012. In vivo architecture and action of bacterial structural maintenance of chromosome proteins. Science **338**:528–531.

4. **Badrinarayanan A**, **Lesterlin C**, **Reyes-Lamothe R**, **Sherratt D**. 2012. The Escherichia coli SMC Complex, MukBEF, Shapes Nucleoid Organization Independently of DNA Replication. Journal of Bacteriology **194**:4669–4676.

5. **Wang X**, **Possoz C**, **Sherratt DJ**. 2005. Dancing around the divisome: asymmetric chromosome segregation in Escherichia coli. Genes & Development **19**:2367–2377.
