# Supplementary figures and images for: The SMC Complex MukBEF Recruits Topoisomerase IV to the Origin of Replication Region in Live Escherichia coli
Source: mBio. 2014 Feb 11;5(1):e01001-13. doi: 10.1128/mBio.01001-13 (PMC3950513; doi:10.1128/mBio.01001-13)

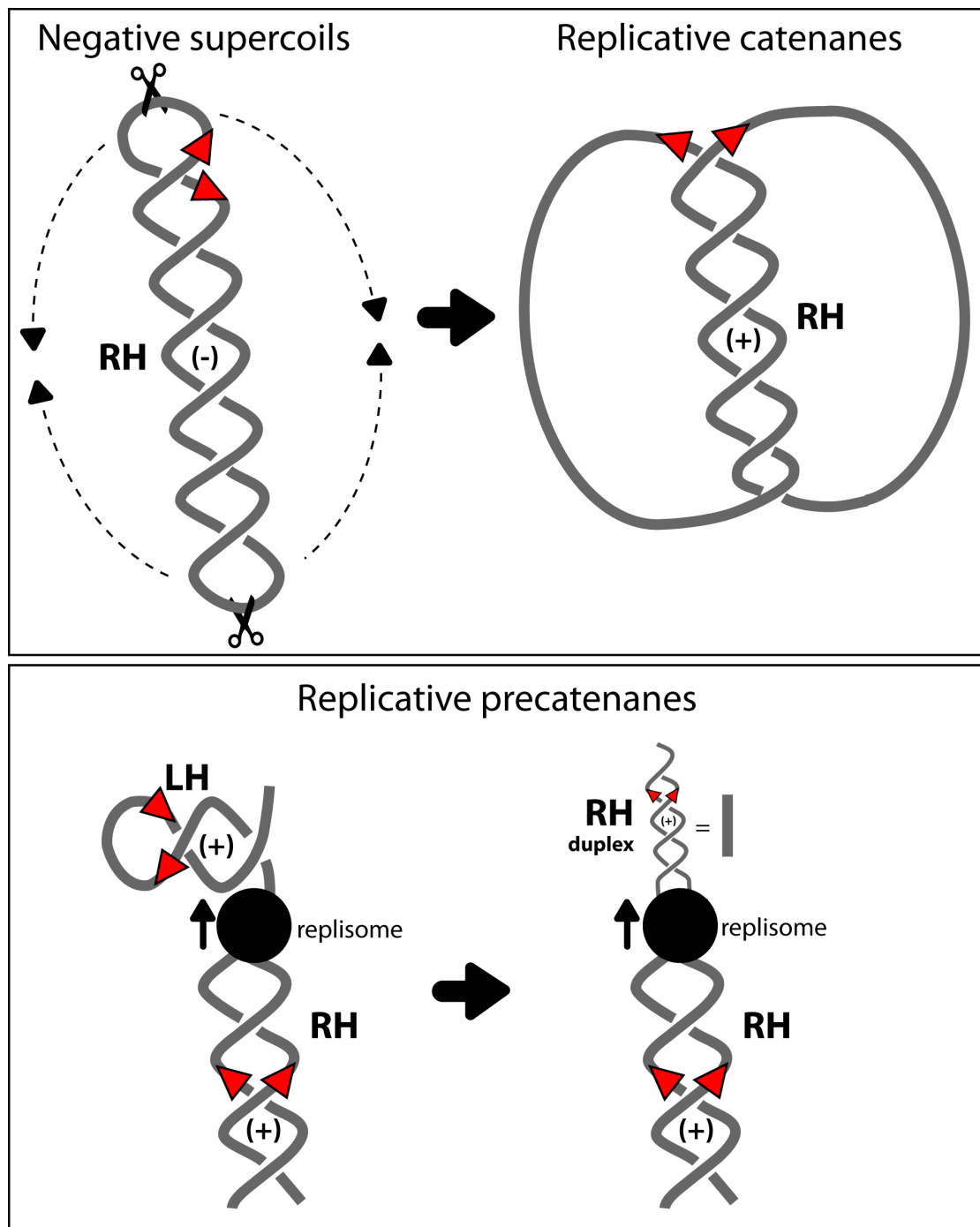

Fig. S1 Nicolas et al.

Supplement: Figure S1 — Topology of replicative (pre)catenanes. Duplex DNA has the two strands of the double helix in a right-handed (RH) (+) configuration (bottom panel, right). Precatenanes that interlink the two sister duplexes after replication have the same RH (+) topology (bottom panel). RH (−) supercoils (SCs) have the same RH chirality as (+) precatenanes, whereas the left-handed (LH) (+) SCs that accumulate ahead of a replication fork have the opposite chirality (bottom left panel). Note that one converts an RH (−) SC to an RH (+) replicative catenane by “cutting” the top and bottom of the plectoneme and rejoining (top panel); the sign changes from (−) to (+) because of the relative change in orientation of the red arrows. Hence, conditions that favor the stimulation of RH (−) SC relaxation are expected to stimulate decatenation, since the substrates have the same RH chirality. Download [file mbo001141739sf01.pdf]

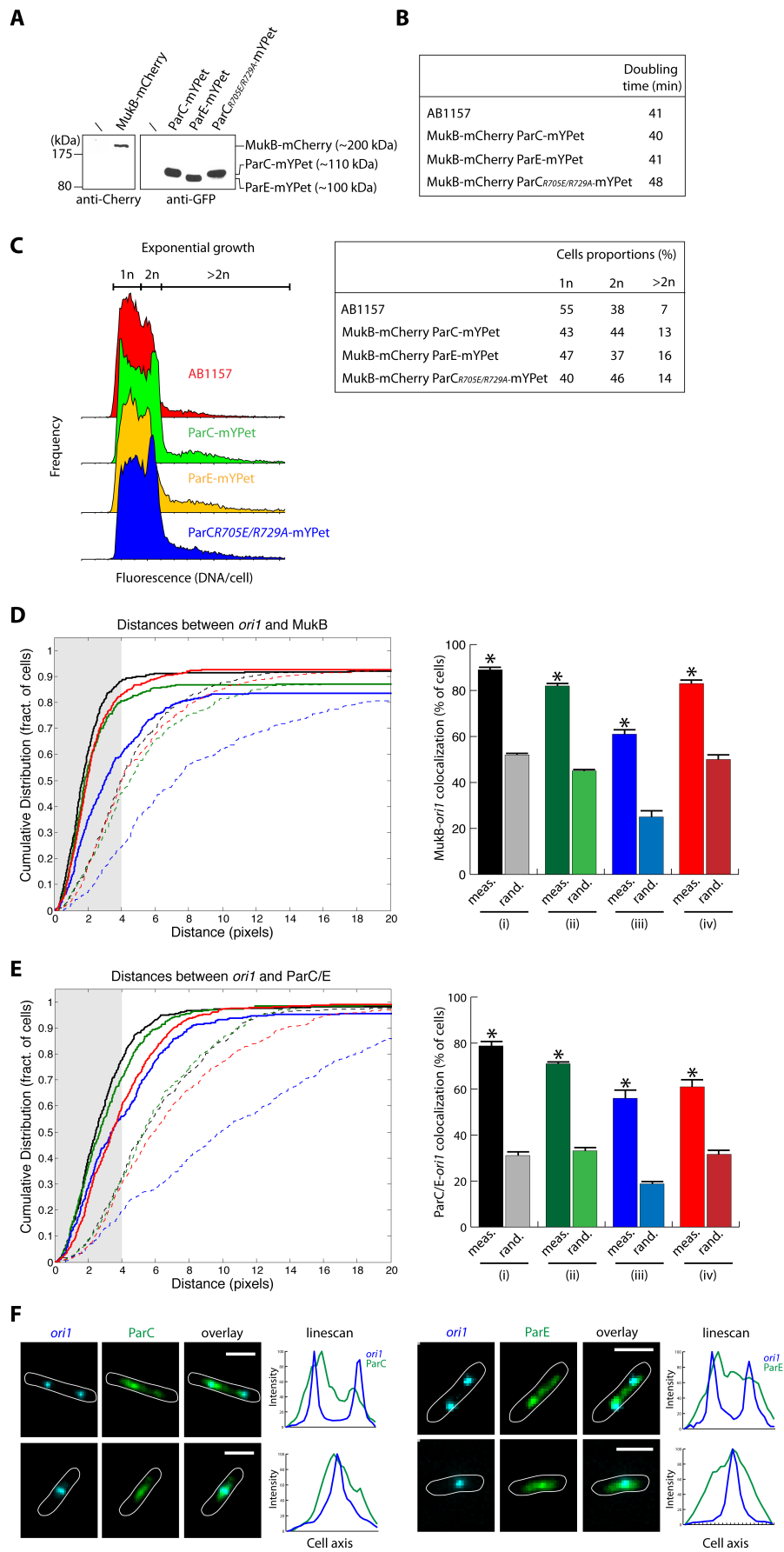

Fig. S2 Nicolas *et al.*

Supplement: Figure S2 — Cell cycle parameters of fluorescent fusions and the demonstration that TopoIV subunits colocalize with MukBEF foci and ori1. (A) Western blot analysis showing the expression of the full-length versions of the different fusion proteins. (B) Generation times of the strains expressing the different fusion proteins. Cells were grown in LB medium at 30°C. (C) Flow cytometry analysis of cells grown in M9-glycerol medium at 30°C. Exponential-phase cells were analyzed. Strains expressing various versions of ParC/E-fluorescent protein fusions were compared to the AB1157 strain. The table shows the different proportions of 1n, 2n, and >2n content (1n includes chromosomes up to ~50% replicated, while 2n includes chromosomes that are >50% replicated). (D and E) Colocalization events between ori1 versus MukB (D) and ori1 versus ParC/E (E). Cumulative curves and histograms were generated as described in the legend to Fig. 1. In panels D and E, black and gray bars correspond to the MukB-mCherry ParC-mYPet ori1-tetO (TetR-Cfp) strain (i), green bars correspond to the MukB-mCherry ParE-mYPet ori1-tetO (TetR-Cfp) strain (ii), blue bars correspond to the MukB-mCherry ParC-mYPet ori1-tetO (TetR-Cfp) strain in the absence of replication (iii), and red bars correspond to the MukB-mCherry ParCR705E/R729A-mYPet ori1-tetO (TetR-Cfp) strain (iv). (F) Representative examples of cells expressing ParC-mYPet or ParE-mYPet in strains where the origin of replication locus was identified by the binding of a TetR-CFP protein on a tetO array (ori1). Bars, 2 µm. Download [file mbo001141739sf02.pdf]

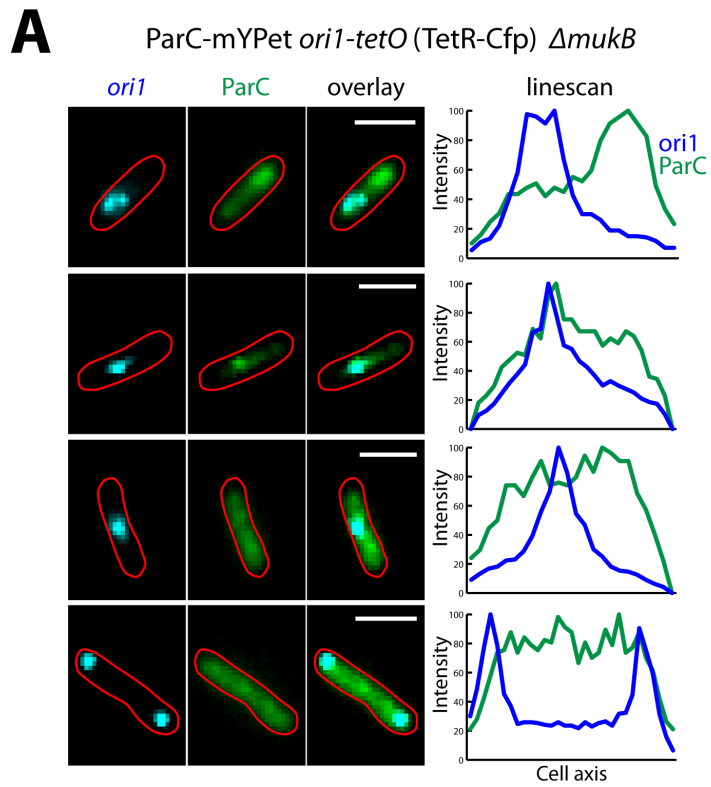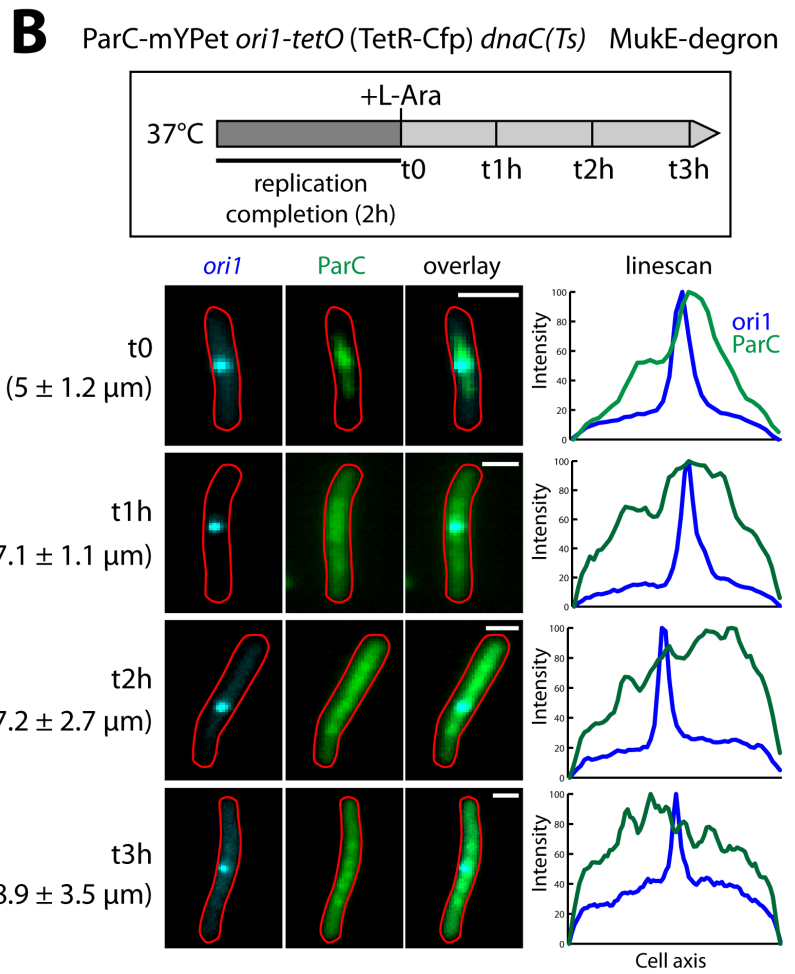

Fig. S3 Nicolas et al.

Supplement: Figure S3 — Deletion of MukB or depletion of MukE leads to loss of ParC colocalization with the origin of replication. (A) Representative examples of cells expressing a ParC-mYPet fusion, with ori1 labeled in a ΔmukB strain. Cells were grown at 22°C in M9-glycerol medium. (B) MukE depletion was performed in the absence of replication [dnaC(Ts) allele at 37°C; depletion started after 2 h at 37°C]. Representative examples of cells at different time points after the addition of l-arabinose (0.5%) are shown. The average sizes and standard deviations of 50 independent cells at the different time points are also indicated. Bars, 2 µm. Download [file mbo001141739sf03.pdf]

**A**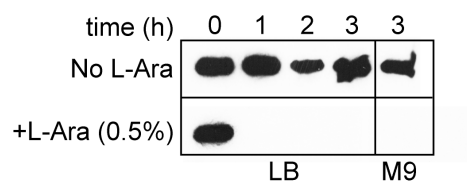**B**+L-Arabinose (0.5%) + *dnaC(Ts)* (37°C)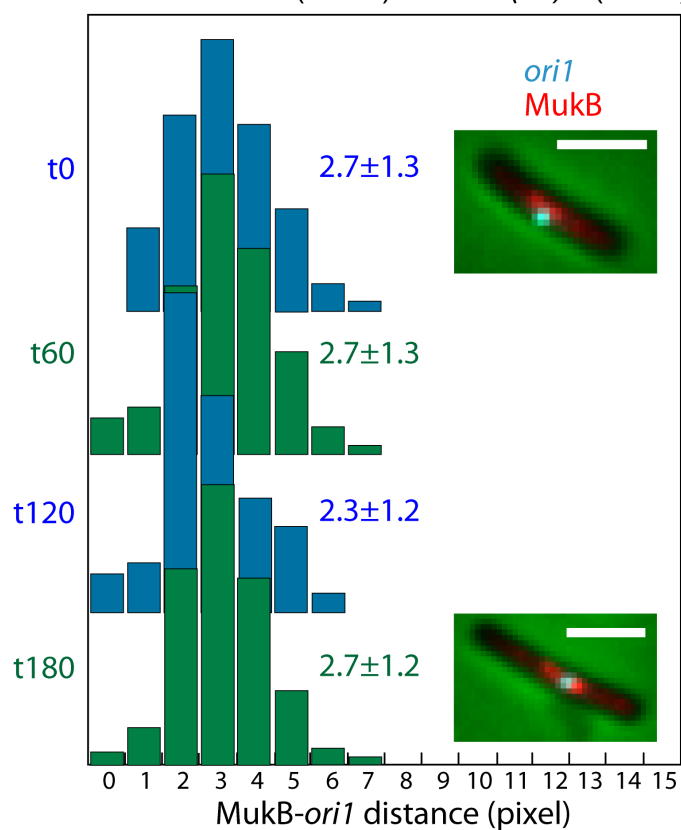**C**

ParE depletion/repletion

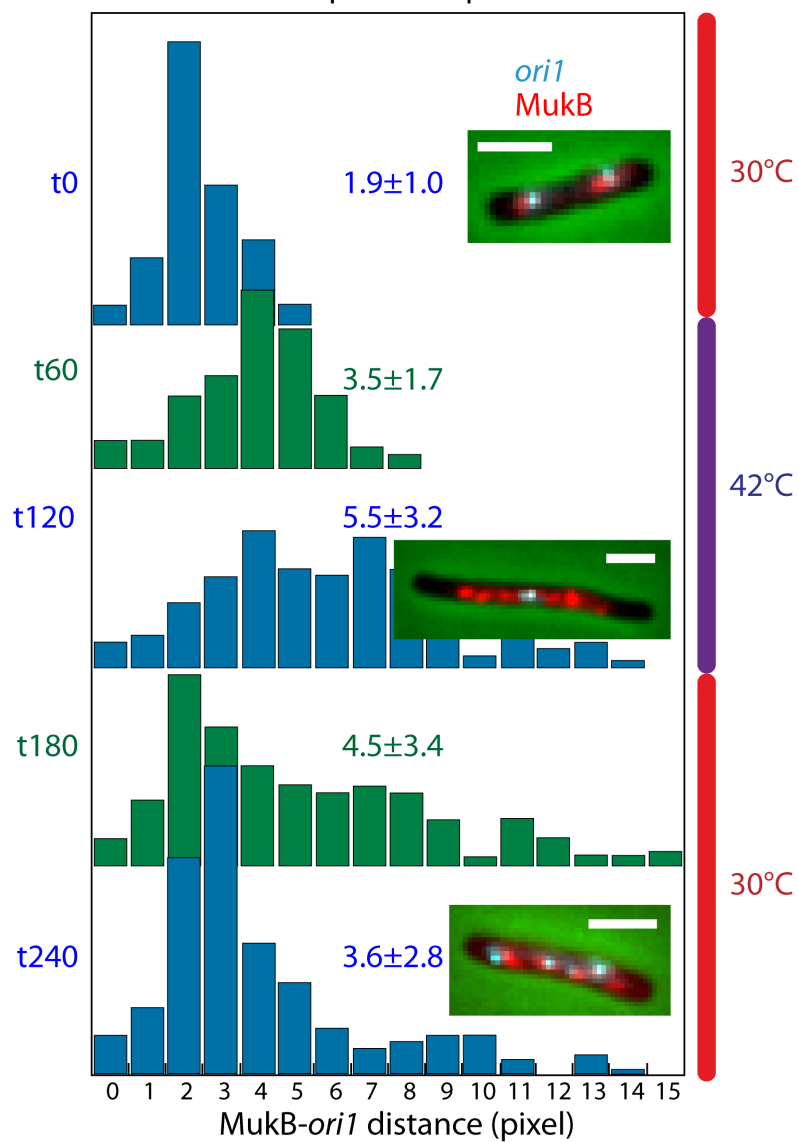Fig. S4 Nicolas *et al.*

Supplement: Figure S4 — MukB foci persist in E. coli cells upon impairment of topoisomerase IV. (A) Western blot showing the disappearance of ParC protein upon the addition of l-arabinose (0.5%) in cultures of E. coli in LB or M9 medium. (B) The histograms show the distribution of MukB-ori1 distances upon depletion of ParC protein in the absence of replication [dnaC(Ts) allele at 37°C; depletion started after 2 h at 37°C]. (C) Impairment of ParE activity was achieved by using a thermosensitive allele of ParE [parE(Ts)] and shift of growth cultures to 42°C. Return to functional ParE was assayed after shift of the cultures to 30°C. Aliquots were taken at different time points, and the distances (in pixels) between MukB foci and the ori1 locus was assessed manually using ImageJ software. Means and standard deviations were calculated. Representative examples of cells at each time point are represented. Bars, 2 µm. Download [file mbo001141739sf04.pdf]

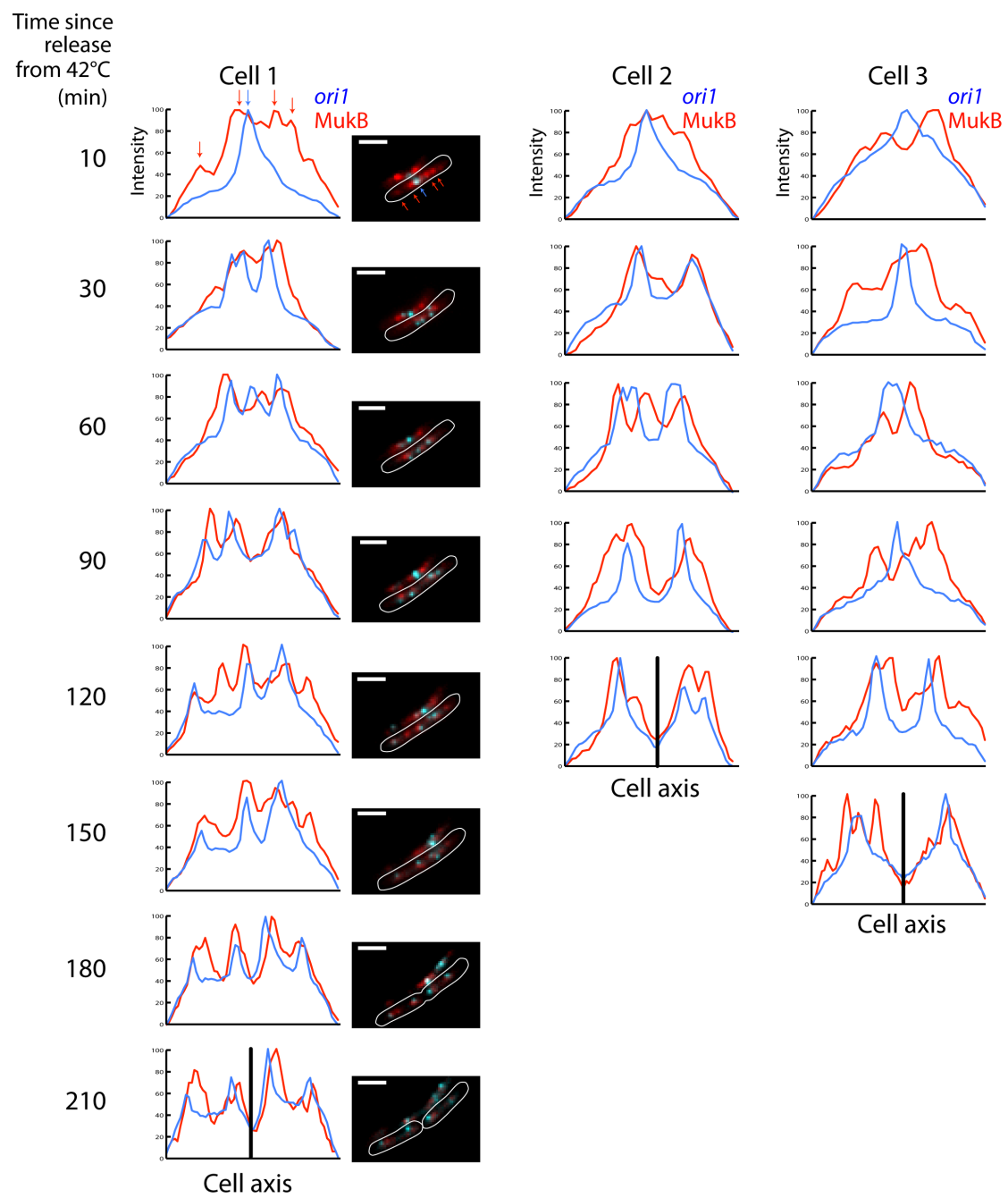

Fig. S5 Nicolas *et al.*

Supplement: Figure S5 — Time-lapse microscopy following ori1 and MukBEF dynamics during the transition from impaired TopoIV to functional TopoIV. Line profile analysis of time-lapse microscopic images for 3 representative cells released from parE(Ts) impairment at 42°C. These are the same 3 cells summarized in Fig. 4. The blue lines represent intensity profiles for ori1, and the red lines represent MukBEF. Cell images are also shown for cell 1. For each cell, the analysis is shown up until the point where the cells had clearly divided; septa are marked by a vertical black line on the graph for the last time point. Download [file mbo001141739sf05.pdf]

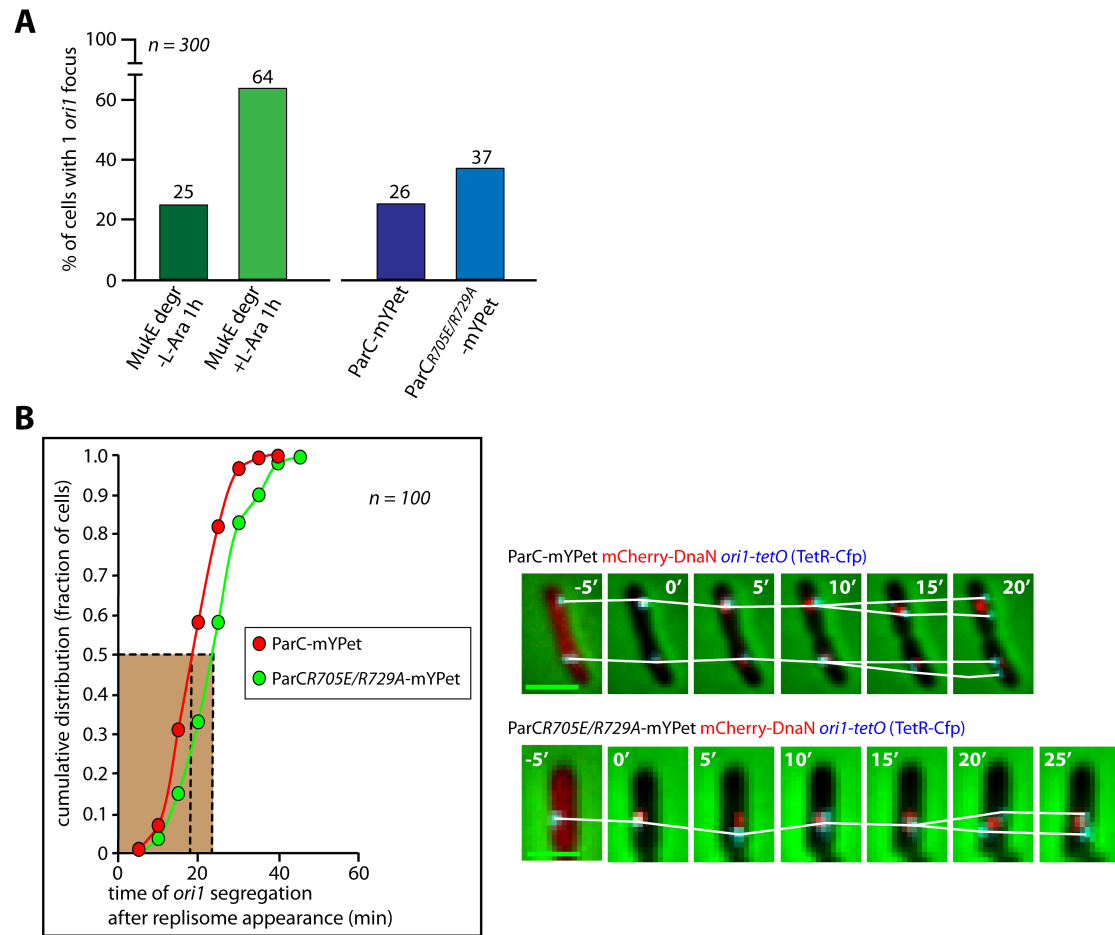

Fig. S6 Nicolas *et al.*

Supplement: Figure S6 — The TopoIV-MukBEF interaction may direct TopoIV-mediated decatenation to ori. (A) Percentage of cells containing a single ori1 locus in indicated strains. (B) Cumulative distributions describing ori1 locus segregation time after replisome appearance (mCherry-DnaN) in strains expressing ParC-mYPet or ParCR705E/R729A-mYPet fusion. Representative examples of such segregation events are shown. Bars, 2 µm. Download [file mbo001141739sf06.pdf]

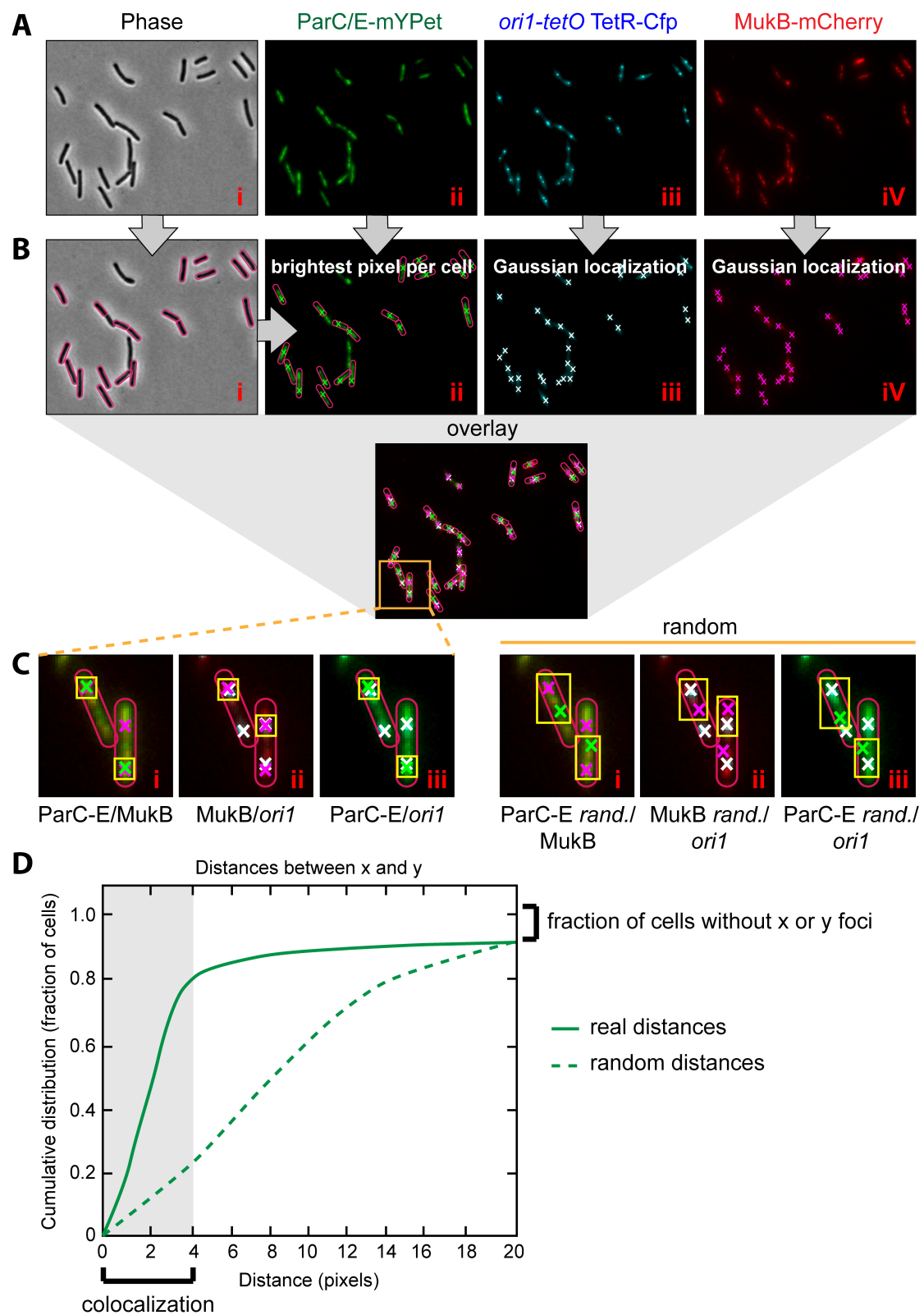

Fig. S7 Nicolas et al.

Supplement: Figure S7 — Analysis of colocalization events. (A) Four different channels were typically imaged during a microscopy experiment: (i) a phase-contrast image, (ii) a fluorescence image in the green channel (GFP filter set) to visualize the mYPet fusion proteins, (iii) a fluorescence image in the blue channel (CFP filter set) to visualize the CFP fusion proteins, and (iv) a fluorescence image in the red channel (mCherry filter set) to visualize the mCherry fusion proteins. (B) Outlines of cells were defined using MicrobeTracker from the phase-contrast image (i), and the generated meshes were used in order to find the brightest pixel by cell in the green channel (ii). Foci assembled by MukB-mCherry (iv) or TetR-CFP (iii) were automatically found using a Gaussian fitting algorithm. (C) Pairwise distances between the closest brightest ParC pixel and the centroids of Gaussian-fitted MukBEF or TetR fluorescent foci were measured (yellow rectangles). Distances between a reference pixel (i.e., from MukB or ori1 focus) and a randomly positioned pixel in the cell were also calculated. (D) The distances were plotted as cumulative distribution curves. Colocalization frequencies were defined as the proportion of cells showing pairwise distances shorter than 4 pixels. Download [file mbo001141739sf07.pdf]
