# Supplementary material for: The SMC Complex MukBEF Recruits Topoisomerase IV to the Origin of Replication Region in Live Escherichia coli
Source: mBio. 2014 Feb 11;5(1):e01001-13. doi: 10.1128/mBio.01001-13 (PMC3950513; doi:10.1128/mBio.01001-13)
Supplement: Table S1 — Plasmids and oligonucleotides used in this study. [file mbo001141739st1.docx]

Table S1 Plasmids and oligonucleotides

| Name | Description | Reference |
| --- | --- | --- |
| pROD10 | pUC18-derived vector containing the cassette 11aaa-mYPet-FRT::Kan^r^::FRT | (1) |
| pROD17 | pUC18-derived vector containing the cassette 11aaa-mCherry-FRT::Kan^r^::FRT | (2) |
| pROD60 | pUC18-derived vector containing the cassette 11aaa-cMyc-DAS+4 degron tag-FRT::Kan^r^::FRT | (3) |
|  |  |  |
| MukB-forward | 5’-aac tcc ctg aaa cgc ttc cag gaa ctg acg aag cgc ctt ctc agg cga gtt cgg ctg gct ccg ctg c-3’ | (2) |
| MukB-reverse | 5’-gaa acg gag ttt tcg gaa aaa gaa aag gcg gca ttg ctg ccg cct taa ttc tta tga ata tcc tcc tta gtt c-3’ | (2) |
| ParC-forward | 5’-gtg ttg aga tcg act ctc ctc gcc gtg cca gca gcg gtg ata gcg aag agt cgg ctg gct ccg ctg c-3’ | This study |
| ParC-reverse | 5’-tcc ggc gtt cct tgc aag cgg gag gaa aca gcg ccc tcc ccg gca tac tta tga ata tcc tcc tta gtt c-3’ | This study |
| ParE-forward | 5’-atc gcc gca act ggt tgc aag aga aag gcg aca tgg cgg aga ttg agg ttt cgg ctg gct ccg ctg c-3’ | This study |
| ParE-reverse | 5’-tcc tgc ctt gtt tgc ccg gcc atc ctg acc ggg caa tgt tct ttc ctc tta tga ata tcc tcc tta gtt c-3’ | This study |
| ParC::mYPet Kan cloning forward | 5’-gca tac atg cat gtc gag ctc atg agc gat atg gca gag c-3’ | This study |
| ParC::mYPet Kan cloning reverse | 5’-gca tac atg cat gtc gga tcc cgc atg aat aaa caa cgg-3’ | This study |
| ParC R705E forward | 5’-cat gtt ggg aaa gag aaa att aaa ctg c-3’ | This study |
| ParC R705E reverse | 5’-gca gtt taa ttt tct ctt tcc caa cat g-3’ | This study |
| ParC R729A forward | 5’-ggt acg ttg atg gcc ggt ttg cag cg-3’ | This study |
| ParC R729A reverse | 5’-cgc tgc aaa ccg gcc atc aac gta cc-3’ | This study |
| ParC mutant integration forward | 5’-atg agc gat atg gca gag cg-3’ | This study |
| ParC mutant integration reverse | 5’-cgc atg aat aaa caa cgg-3’ | This study |

Underlined nucleotides correspond to homologous recombination sites for chromosomal integrations

References

1. **Reyes-Lamothe R**, **Possoz C**, **Danilova O**, **Sherratt DJ**. 2008. Independent positioning and action of Escherichia coli replisomes in live cells. Cell **133**:90–102.

2. **Badrinarayanan A**, **Reyes-Lamothe R**, **Uphoff S**, **Leake MC**, **Sherratt DJ**. 2012. In vivo architecture and action of bacterial structural maintenance of chromosome proteins. Science **338**:528–531.

3. **Reyes-Lamothe R**, **Sherratt DJ**, **Leake MC**. 2010. Stoichiometry and architecture of active DNA replication machinery in Escherichia coli. Science **328**:498–501.
